# Still lost in transition: a qualitative descriptive study of people’s experiences following treatment completion for haematological cancer in Wales, UK

**DOI:** 10.3332/ecancer.2019.985

**Published:** 2019-12-12

**Authors:** Tessa E Watts, Janet Bower

**Affiliations:** 1 School of Healthcare Sciences, Cardiff University, Cardiff CF10 3AT, UK; 2 Chemotherapy Day Unit, Hywel Dda University Health Board, Withybush General Hospital, Haverfordwest SA61 2PZ, UK; a https://orcid.org/0000-002-1201-5192

**Keywords:** qualitative research, haematological cancer, supportive care needs, unmet needs, patient experience

## Abstract

The aim of this study was to explore Welsh adults’ experiences of the transition into survivorship from initial active systemic anti-cancer treatments for haematological cancers. An exploratory, qualitative descriptive study consisting of in-depth, face-to-face interviews was designed. A convenience sample of adults in Wales, UK, who had completed their initial systemic anti-cancer treatment for haematological cancer was recruited from one University Health Board. Data were generated in digitally recorded, individual, face-to-face interviews during 2017. Interviews were fully transcribed and analysed using a qualitative thematic approach.

Seven people participated in interviews. Thematic analysis revealed three themes: encountering ambiguity, the pursuit of normality and navigating treatment completion. The transition from patient to survivor was characterised by trepidation and uncertainty. While participants sought to resume a sense of normality in their lives, they were beset by enduring treatment effects. They felt insufficiently prepared for these effects and were uncertain about the availability of the ongoing supportive care which met their individual needs. Participants articulated that they desired much more from haematology providers in preparing them for life beyond initial SACT.

## Trial registration

The study was approved by NHS Research and Ethics Committee [Project ID 226961] on 28 May 2017.

## Background

Internationally, research has consistently shown that adults affected by haematological cancers have unmet needs across their illness trajectories [1–7]. Described as a mismatch between what individuals perceive as necessary to optimize their health and the care and services actually received [1], unmet needs can impact negatively on health, wellbeing and quality of life. Most studies conclude that people affected by haematological cancers are underserved, and that to enhance survivorship outcomes, supportive care interventions and care coordination must be improved [5, 6, 8–10].

While some newer haematological cancer treatments are less toxic [11], others are complex, intense, debilitating and burdensome [6, 12, 13]. Treatment completion, also known as re-entry [14, 15], is a transitional point in cancer trajectories. The end of treatment may be eagerly awaited and, when it arrives, marked by joy juxtaposed against relief. However, life may have changed and the known, trusted and reassuring safety net of treatments and the attendant professional and peer support may have been cast away [10] There may be expectations, on several levels, of resuming a normal life. Nevertheless, debilitating physical and/or psychological problems, for example, severe fatigue, persistent anxiety and uncertainty, can endure or even develop long after treatments cease [16–19]. Furthermore, individuals may experience altered role and physical functioning, financial and employment concerns and relationship difficulties [1, 16]. With no clearly defined ending to their illness in sight and viewed through the lens of the theoretical concepts of liminality [20] and biographical disruption [21], individuals’ realities on initial treatment completion may be that they are caught between the binary of illness and wellness, ‘betwixt and between’ and living in an unknown, ambiguous and liminal place.

International research has documented the myriad of challenges experienced, often unexpectedly, in the aftermath of initial cancer treatment [22, 23]. These include feeling unprepared [24–27], experiencing uncertainty [24, 26, 28], fear of recurrence [24, 26, 28, 29] and being insufficiently informed about late treatment effects [24, 26], self-management strategies and local support services [24]. However, for people affected by haematological cancers, this distinct point in the illness trajectory is still poorly understood, for it remains relatively unexplored. Yet the experiences of and issues for people living with and beyond haematological cancers are increasingly relevant, not least because their incidence and prevalence are steadily rising globally [30, 31]. While clinical outcomes vary across the numerous haematological cancer sub-types [30, 32, 33], the UK has 44,390 new cases annually, 5-year prevalence is 163,800 [11] and survival across most of Europe is improving [34]. The population of people living with and beyond haematological cancer is therefore growing, a trend which will continue with ageing, improving detection and treatment advances [12, 34]. Better understanding of individuals’ experiences in the immediate aftermath of treatment completion is required to provide insight and information to guide person-centred service enhancement. Accordingly, this paper reports the findings from a qualitative study conducted in the devolved nation of Wales, UK which sought to obtain in-depth understanding of adults’ experiences of their transition from initial active treatment for haematological cancers into survivorship.

## Method

As this was an exploratory study, a qualitative descriptive design [35] using in-depth interviews and thematic analysis was adopted. The study was conducted in Wales, UK. Following approvals from National Health Service and health board research ethics and governance, individuals with haematological cancers who had completed initial active anti-cancer treatment were recruited. Adults meeting the inclusion criteria (Table 1) were identified by oncology outpatient clinicians, and given a study pack comprising an invitation letter, study information sheet and response slip to return directly to the researcher (JB) if they were interested in taking part.

With participants’ consent, in-depth, face to face, digitally recorded interviews were conducted in 2017 in a location of participants’ choosing. Interviews have a long history in qualitative research and are used extensively to generate rich data that confers insight into experiences, thoughts, feelings and interpretations [36]. Interviews were aided by a loose interview guide (Table 2) derived from the literature and consultation with service users.

Interviews were fully transcribed. To protect anonymity, all participants were allocated a unique pseudonym known only to the researcher. Transcribed data were analysed using an analytic framework [37] widely used in the qualitative research. This systematic approach involves searching for patterns of meaning across the dataset. Transcripts were repeatedly read to ensure data familiarisation and then manually coded. Codes were abstracted into sub-categories, categories and broad overarching themes and scrutinized for similarity and duplication.

Trustworthiness was enhanced by drawing on measures to achieve credibility, transferability, dependability and conformability [38]. Meticulous transcription and checking of interviews and rigorous data analysis by two researchers independently contributed to credibility. Detailed descriptions of experiences aided transferability while an audit trail of methodological decisions ensured dependability and confirmability.

## Results

Eight people expressed an interest in participating in the study and seven were interviewed: three women and four men, who were between 8 months and 3 years post-active treatment. Two participants had undergone stem cell transplant while others had received either chemotherapy alone or in combination with Rituximab. The interviews lasted between 35 and 45 minutes.

Three key themes were identified from data analysis: *‘Slightly unsure’?*: Encountering ambiguity; *‘Trying to get back to my normal’:* the pursuit of normality*; ‘What happens now’?:* navigating treatment completion.

### Slightly unsure: encountering ambiguity

Treatment completion was a celebrated milestone for it marked an important juncture in participants’ cancer journeys. However, while it was met with thanks, gladness and relief, there was also a deep sense of trepidation. Indeed, cast adrift from the surveillance and relative safety of the specialist centre with its intensive professional and peer support, participants were uneasy about their future, not least because they were concerned their cancer could return.

Participants had welcomed treatment completion for, as Annie explained, during treatment *‘you just feel like it’s the end’.* This eagerly awaited milestone meant the cessation of intensive, debilitating and for some even ‘*traumatic*’ interventions:

*I was praying for the last one* [treatment] *to come along (….). A sense of relief I guess it was that it* [treatment] *was over. (…) I had got to the stage where I was dreading coming up.* (…) (Christopher)

However, there was a sense of loss at leaving the treating team’s safety net of constant surveillance and the supportive bonds cultivated therein. The magnitude of loss is encapsulated in the metaphorical language in the following data excerpts:

*You are in this umbrella and you want to stay here and see people here.* (Christopher)

*I felt I lost part of me like, cos I look upon, up here as a part of your home, not a home, but a group, somebody to talk to, same conditions as you.* (Gareth)

Despite their supportive families, many participants felt *‘alone and lost’* (Annie) and extremely vulnerable at this juncture. There was a palpable sense of insecurity and enduring ambiguity about the future. The possibility of recurrence or relapse and awareness of mortality permeated participants’ accounts. Some openly expressed these anxieties:

*It’s always in the back of your mind. I get a bit depressed, and I think is it going come back? When’s it going to come back? How long have I got?* (Gareth)

*You begin to wonder, is this the end or is this the start of another session of it?* [cancer]. (Bill)

For others, anxiousness about possible recurrence or relapse was manifest in the vocabulary that cancer was ever present:

*It’s there somewhere in my body it’s probably a little cyst or something.* (Debbie)

*I think it’s a thing at arm’s length at the moment. It’s in me but it’s under control.* (Christopher)

### ‘Trying to get back to my normal’: the pursuit of normality

Despite a prevailing sense of ambiguity, when treatment ended participants prioritised recovery and a return to normality. Yet there were limits to what participants could realistically and comfortably achieve. To find a way through participants drew on different self-management strategies, and through a process of acceptance and readjustment, their normal was redefined.

Participants’ accounts indicated that treatment completion signalled time for recovery and attempting to restore and maintain a sense of normality:

*Trying to go back to normal and do what you normally do.* (Annie)

They spoke of resuming everyday activities, routines, hobbies and taking holidays. For those who were employed (*n* = 2), returning to work was important, for it would provide structure, support and financial stability. Nevertheless, workplace reintegration was contingent on supportive, facilitative and flexible employers:

*They* [employer] *put me on a phased return which was over a period of eight weeks, and I started doing 4 hours a day, for two weeks then 5 then 6 then 7, so it was over about ten weeks. And I am back to full time now.* (Christopher)

In contrast, Annie described her *‘shock’* and heartbreak at discovering that after treatment she was unable to return the job she *‘loved’.* Her distress was amplified by the felt stigma of being reliant on state welfare, her perception of *‘scrounging’* and the haunting of the humiliating words that she was no longer *‘fully functional’.*

*To say you are not fully functional is like a bombshell, it’s like a punch in the face. That was hard and I don’t think I will ever get to terms with that.* (Annie)

This experience left an indelible mark on Annie’s sense of self and identity. Yet despite her heartache, Annie described how she tried to play down the impact of what was happening by projecting an aura of normality:

*Trying to put a brave face on things and people around you. You try not to affect them or hurt them. And that’s hard. And to be positive. That’s the hardest thing I have ever done. (…). My daughter is with me all the time and I pass things off oh ‘I’ll be fine’ you know but deep inside you are suffering like anything.* (Annie)

Annie’s words convey she engaged in hard work, regulating and managing her emotions to protect her family. The work of constructing an outward image of normality in the face of adversity and precariousness was not unusual. The words *‘I am fine’,* conveyed a finely veiled message of normality throughout participants’ accounts. Sometimes, however, participants chose to remain silent:

[I] *keep it to myself, I wouldn’t say much to my wife.*

**Is that a conscious decision?**

*Yeah I think it is, because I don’t know. I don’t want to burden and worry people, because I know my wife, she would worry about anything.* (Harry)

Constructing an outward image of normality enabled participants to maintain a sense of control. Yet after treatment ended unpleasant, debilitating treatment effects lingered on or even emerged. Mainly, these effects were physical and psychological and included fatigue, nausea, insomnia, anxiety and low mood. An *‘overriding feeling of not being well’* prevailed, imposing unanticipated enduring disruption in participants’ lives:

*I couldn’t even climb the stairs, no energy, no nothing. Couldn’t even dress, wife had to undress me, shove me in bed. Climb halfway up the stairs, sit on the step,10 minutes up to the top step, stop for ten minutes, sit on the bed.* (Gareth)

*I wasn’t buoyant anymore (….) I got a little bit down and I think I was depressed, and there was all sort of niggly bits starting I didn’t I didn’t pick up my tai chi sport, (….) I was beginning to get isolated. I would only go out with my husband and didn’t go off on my own like I was used to.* (Ffion)

Against this background and to facilitate recovery, adjust and restore some semblance of a normal life, participants drew on different self-identified self-management practices contingent with their needs, goals and understandings. Some turned to positive lifestyle modifications in terms of smoking cessation, reduced alcohol intake, physical activity and improved diet:

*I do the exercise they told me, I stand up and do the standing up press ups. (….). I don’t know how many stairs I have done today? I’m useless on this bloody watch (…), 15 floors. Sometimes I do 50 odd. And what steps? 5000 steps.* (Harry)

*I try to keep myself healthy by eating healthy. (….). I probably eat a bit differently I think, because now I have cereal and yoghurt in the morning (…)and I don’t eat so much junk food. Because you think it’s important to try and keep your body healthy.* (Annie)

To mitigate worry, online forums, third sector support were accessed:

*I joined a lymphoma society online (…) and I found that a godsend, there is a hell of a lot of information on that and you just type in, there’s forums there, there are people there with the same condition as you, people with the same problems.* (Gareth)

Participants also reappraised their lives, priorities and relationships for their illness had made them think about what was important, what mattered and living in the moment:

*Now I think no, this is my life and I have got to live it the way I want to live it.* (Ffion)

I want to get everything done, now, because you don’t know how long before it comes back, if it comes back. (Gareth)

Thus, an enduring sense of illness meant that through adjustment and acceptance, normal was redefined and gave way to a new normal:

*You’ve got to learn to live with and cope with it (…). Try to do things that I normally do but you know I can’t always do everything that I want to do but I do try.* (Annie)

### ‘What happens now?’: navigating treatment completion

Participants had felt unprepared for the enduring treatment effects and isolation experienced in the aftermath of treatment completion. They identified a pressing need for enhanced continuing supportive care, preferably, but not exclusively provided by the specialist treatment team.

Some were prepared to tolerate the problems they were experiencing for they had been left with the gift of life and a second chance. However, insufficient specific preparation for enduring and late treatment effects threaded through participants’ accounts:

*You would walk. You would go up a path and up another path, and I was having to stop twice and I thought what the hell is going on here? (….). I did talk to them* [doctors] *about it. He* [doctor] *told me that chemotherapy can attack organs in your body. Well I said, no-one told me that.* (…) *I would have liked for someone to say, you could have problem with some of your organs in your body.* (Harry)

*In the beginning* [of treatment] *the set up was wonderful and* [nurse] *gave me all these things and the information and the phone numbers in case things went wrong (….). But there was not much afterwards, information. That I might feel depressed or down, can’t sleep, I mean worrying.* (…). *I didn’t know what to expect.* (Ffion)

Participants felt ongoing surveillance by their haematology doctors via routine medical follow-up clinics was reassuring. They were acutely aware that clinics were extremely busy places and were immensely grateful to be seen. However, they felt the quality of the consultation was limited for it was rushed, biomedically driven and doctor led and primarily focused on reviewing blood results:

*I know there is a lot of people to see but I think it could do with somebody with a bit more time with you. To say how you are feeling, because when you come for an appointment here it’s sort of ‘how are you today?’ blah blah and that’s it and you are out.* (Annie)

Participants felt that the physical and psychological issues they were experiencing were perceived to be of secondary importance and thus held in abeyance:

[Doctor] *is very to the point, and I found if you spoke about anything else at times you would almost feel you were in the wrong for asking that type of questions.* (Christopher)

While participants spoke affectionately about their general practitioners, there was uncertainty as to whether they were suitably positioned to meet their needs. Indeed, participants described the busyness, lack of specialist knowledge and discontinuity in GP services:

*They* [GP’s] *are overworked as well. So, do they really understand the in-depth of what’s going on within the body?* (Ffion)

*The trouble is now we’ve got this system where you go in and you don’t know who you gonna see. With a regular one* [GP] *you could talk to them (…).* (Debbie)

All participants articulated the need for enhanced supportive care after treatment. While views on the precise nature of the desired supportive care varied, the prevailing viewpoint was that it should be provided by the specialist treatment team. This was because participants trusted and had confidence in the team’s knowledge, skills and expertise:

*The first thing I would do is get in touch with you lot up here you. You lot know what you are doing and I would think I want to start there. I could start at the doctors but all he would be doing is referring to you, I think, I would want to go straight to the horse’s mouth to the people who are supposed to know.* (Bill)

At the same time, high value was placed on peer support, speaking with others who had also been through the treatment experience, either on one-to-one or group basis:

*A support group would be a good thing where they can talk about how they feel in front of other people because I think people are probably more open if that was there, and you think ‘oh actually’ and then you can help someone else as well.* (Annie)

Participants also described a need for better information on treatment completion and made their own suggestions on what could be valuable in supporting the transition into survivorship:

*Is there a possibility that they* [patients] *can have some information that you can give them? Some little bit and you can say ‘that’s what you can do?’* (Ffion)

*It’s quite easy to get a piece of paper and you put it in a folder or whatever and you just forget about it until you need it.* [People] *should be aware (…) bits that you need to really know.* (Annie)

## Discussion

The findings from this interview study revealed that on treatment completion, participants encountered enduring, unanticipated disruption in their lives. They felt unprepared for the transition and treatment effects and, although self-management strategies were embraced, there was a perception of inadequate continuing professional support. This study adds to the limited existing literature describing the experiences of adults who have completed the initial treatment for haematological cancers at what is a key transitional point. The findings have value as they offer specialist haematological staff rich insight into the challenges people encounter on the completion of the initial treatment, their unmet supportive care needs and why and how they feel these may be addressed. The findings are important because they unveil that people with haematological cancers are still insufficiently prepared for life beyond treatment. Furthermore, there is a knowledge deficit in terms of available support. Accordingly, the findings will be of worth in driving and supporting innovation in high quality, individualised, person-centred service provision for survivorship care at a critical juncture.

The strength of this study is the generation, through in-depth interviews, of rich, detailed narratives which encompass the breadth of individuals’ unique experiences at this key transitional point in their cancer journey. Nevertheless, the study has limitations for it reflects insights of a small, heterogeneous sample of self-selecting participants at one time point and in one geographical area. Yet the sample size is not a methodological limitation for it is in accord with exploratory qualitative research and in terms of meeting the study’s aims adequate. Furthermore, findings are not intended to be representative and generalized to the wider population. While recall accuracy must be considered, insights are valuable, have commonality and connect with findings from the previous research.

Participants’ vocabulary affirmed they welcomed initial treatment completion. While treatment had been traumatic and disrupted their lives, this had been mediated by the intense, continuous surveillance and support of the specialist treatment team. Follow-up notwithstanding, discharge meant this level of surveillance ended. The magnitude of the personal sense of loss experienced at this point is made visible in participants’ metaphorical language and the implication that without treating team’s support they felt different, fragmented and no longer whole.

As in previous studies [4, 29], data revealed that treatment completion generated disruption and concern. Concerns about the possibility of relapse or recurrence and awareness of mortality cast lasting shadows. In the absence of intensive specialist surveillance and reflecting findings from the previous studies [7, 39, 40], this concern was a disruptive force. It rendered participants vulnerable, emotionally and existentially, and engendered a profound sense of ambiguity about their futures. There is a sense in which the disruption they experienced constituted a biographical disruption, for their life, hopes and self-concept had altered [21]. Furthermore, being shrouded in ambiguity and drawing on the anthropological work of van Gennep [41] and Turner [20], participants found themselves in a liminal state for they were betwixt and between the binary of being ill and being completely well.

Participants’ everyday lives had been fractured by cancer and its punishing treatment. In the liminal space of treatment aftermath, and consistent with the previous research [42], active pursuit and resumption of normality were core priorities. Participants’ accounts indicated that they exercised agency and worked hard to resume routine activities, family and work responsibilities, a finding which resonates with those from earlier studies [42–45]. Attempting to assert some continuation with the biographical trajectory of life before illness may be connected with societal expectations and, thus tacit pressures, to resume normal life [46]. Furthermore, resuming routine activity is an important phase in recovery, a powerful symbol of normality and may convey a ‘restored self’ [47]. Yet the data revealed that attempts to resume normal life were not always successful. The inability to meet self and others’ expectations could shatter an individuals’ sense of self and, drawing on Bury [21], sustain biographical disruption. Even so, despite the underlying disruption and resonating with findings from previous studies [48, 49], some individuals engaged in difficult emotion work to maintain an external aura of normality and protect themselves and those they loved.

The myriad of unanticipated, enduring treatment effects, which did not always subside with the passage of time, had a profound impact on participants’ lives, general sense of well-being and thus normality. To reclaim a sense of normality and self and reflecting findings from earlier studies [48], participants were motivated to practice self-management to promote and enhance their recovery. Self-management was perceived as a way of reinstating a sense of control of their health and physical and psychological well-being and reintegrating with their social world. The quest for normality is consistent with findings from the previous studies [29, 42, 48]. Yet being well, yet unwell, disruption persisted, and while normality was reconstructed and redefined, participants remained caught in a liminal space.

The complex, potent amalgamation of psychological suffering, enduring physical symptoms and the impaired role and social functioning signals the need for and importance of proactive, person-centred supportive interventions in the immediate post-treatment phase. Indeed, the Institute of Medicine [50] noted the criticality of the transition from the treatment to post-cancer treatment phase. Nevertheless, our data revealed that while participants valued scheduled, consultant led follow-up and held their general practitioners in high regard, many still felt cast adrift, isolated, vulnerable and generally unsupported by the healthcare system. This is consistent with the findings from the previous studies [2, 51] and to some extent relates to the biomedical as opposed to the experiential person-centred focus of regular follow-up and acknowledgement that general practitioners do not have specialist knowledge.

Unlike findings from an earlier study [42], participants wanted some form of ongoing personalised support to enable them to live with the everyday challenges they faced. An unexpected finding was that while support was available locally, our data revealed that participants were unaware of this. At the first sight, this may be read as suggesting difficulty while accessing information and navigating the system. However, an alternative reading, drawing on the work of Swash *et al* [2], is the possibility that existing services were designed around the totality of cancer support services and, therefore, perceived by our participants as irrelevant.

The findings signal and reinforce the importance of embedded, systematic and proactive approaches to care in the shape of integrated, person-centred recovery packages. These interventions may enable people to begin to plan for and navigate the unknown territory of life in ‘*remission society’* [52] and begin to make a positive difference to patient-reported outcomes in terms of long-term quality of life and well-being. Through early identification of holistic needs, information sharing, coordination and supported self-management, people in the post-initial treatment phase may be activated and empowered to become proactive partners in care: planning and using individual strategies which will enable them to optimise and maintain their health, well-being and quality of life.

## Conclusions

Our findings suggested that on treatment completion, participants experienced a lasting struggle for they had unmet supportive care needs and were uncertain of where to turn. They desired much more in the way of ongoing supportive care. Haematology providers have a critical role to play in preparing these people for life beyond their treatments and ensuring that they are cognizant of potential long-term and late effects, supportive care and different self-management strategies. To ensure that people are not lost in transition, specialist nurses working across care boundaries are well placed to have important and influential roles in facilitating recovery in terms of building self-efficacy, ensuring continuity and coordination of care and enabling access to services. Nevertheless, empirical investigation is required to capitalize on the findings reported here.

## Conflicts of interest

The authors declare that they have no conflicts of interest.

## Authors’ contributions

TE Watts co-designed the study, analysed the data and drafted the manuscript.

J Bower co-designed the study, collected and analysed the data and contributed to drafting the manuscript.

## Funding declaration

This study was supported by Tenvous and Health and Care Research Wales through the Research Capacity Building Collaboration (RCBC) First into Research scheme.

## Figures and Tables

**Table 1.** Inclusion and exclusion criteria.

**Inclusion criteria**	Over 18 years of age. Diagnosed with and treated for a haematological malignancy. At least 6 months post initial SACT completion. Willing to participate and be interviewed in the English language. Able to give informed consent.
**Exclusion criteria**	Unwilling or unable to participate. Still receiving SACT. Unable to give informed consent.

**Table 2.** Loose interview guide.

Interview guide
Discussion of experiences of treatment. Discussion of feelings and experiences on completion of treatment. Discussion of experiences of follow-up and recovery, including support systems accessed. Views on enhancing the organisation and delivery of supportive care post initial treatment. Anything else that they would like to share. Clarification will be sought as necessary to enrich the descriptions
